# Cross-Sectional Analysis of the Association between Periodontitis and Cardiovascular Disease Using the Korean Genome and Epidemiology Study Data

**DOI:** 10.3390/ijerph17145237

**Published:** 2020-07-20

**Authors:** Soo Hwan Byun, Sunki Lee, Sung Hun Kang, Hyo Geun Choi, Seok Jin Hong

**Affiliations:** 1 Department of Oral & Maxillofacial Surgery, Dentistry, Sacred Heart Hospital, Hallym University College of Medicine, Anyang 14068, Korea; purheit@daum.net; 2 Research Center of Clinical Dentistry, Hallym University Clinical Dentistry Graduate School, Chuncheon 24252, Korea; 3 Division of Cardiology, Department of Internal Medicine, Dongtan Sacred Heart Hospital, Hallym University College of Medicine, Dongtan 18450, Korea; galiard@hallym.or.kr; 4 Department of Biomedical Sciences, College of Medicine, Hallym University, Chuncheon 24252, Korea; malice23@nate.com; 5 Hallym Data Science Laboratory, Hallym University College of Medicine, Anyang 14068, Korea; 6 Department of Otorhinolaryngology-Head & Neck Surgery, Sacred Heart Hospital, Hallym University College of Medicine, Anyang 14068, Korea; 7 Department of Otorhinolaryngology-Head & Neck Surgery, Dongtan Sacred Heart Hospital, Hallym University College of Medicine, Dongtan 18450, Korea

**Keywords:** periodontal diseases, risk factor, periodontitis, cardiovascular disease, stroke, ischemic heart disease, inflammation, oral health

## Abstract

This cross-sectional study aimed to evaluate the association between periodontitis and cardiovascular disease (CVD) by reviewing and discussing the role of the oral microbiome in periodontitis and CVD. This prospective cohort study used epidemiological data from the Korean Genome and Epidemiology Study from 2004 to 2016. We selected 9973 patients with periodontitis and 125,304 controls (non-periodontitis) from 173,209 participants and analyzed their medical histories to determine the relationship between cerebral stroke/ischemic heart disease and periodontitis. The participants were questioned about any previous history of hypertension, diabetes mellitus, hyperlipidemia, cerebral stroke (hemorrhagic or ischemic), ischemic heart disease (angina or myocardial infarction), and periodontitis. Their body mass index, smoking habit, alcohol intake, nutritional intake, and income were recorded. The Chi-square test, independent *t*-test, and two-tailed analyses were used for statistical analysis. The adjusted OR (aOR) of periodontitis for stroke was 1.35 (95% confidence interval (CI) = 1.16–1.57, *p* < 0.001). The aOR of periodontitis for ischemic heart disease was 1.34 (95% CI = 1.22–1.48, *p* < 0.001). We concluded that periodontitis was associated with CVD and may be a risk factor for CVD. However, further studies are required to determine the association between periodontal treatment and CVD.

## 1. Introduction

The term human microbiota refers to the microorganisms that reside on or within the human body, and the human microbiome (which is the aggregate of all the microbiota) contributes to both health and disease [1,2,3]. In health, the vast bacterial microbiome is a key component in the development of the mucosal barrier and the innate and adaptive immune systems [2,3,4]. Commensal microbiota can cause chronic inflammation and immune disturbances in diseased states, owing to the disruption of the mucosal barrier, while dysbiosis of the microbiota is associated with the development and exacerbation of diseases such as cardiovascular disease, type 2 diabetes, obesity, and inflammatory bowel disease [1,5,6,7]. The development of culture-independent bacterial DNA sequencing techniques can aid in the identification of the characteristics of this vast microbiome and abnormalities in the microbiota; hence, several researchers have been interested in the effects of microbiome dysbiosis on the immune system [2,3].

According to the World Health Organization report, 17.9 million people die each year from cardiovascular disease (CVD) [8], with 3.9 million deaths (45% of deaths) in Europe alone. While the mortality rate of CVD has decreased, the number of deaths associated with it has increased over the last 25 years, owing to the aging of the populations of several countries [9]. CVD is a comprehensive term that encompasses coronary heart disease, atherosclerotic diseases, cerebrovascular disease, and peripheral vascular disease. Atherosclerosis is a chronic inflammatory disease that is associated with risk factors that are responsible for the oxidative and inflammatory phenotypes of this disease [10]. The balance between the stability and instability of the atherosclerotic plaque induced by cellular apoptosis, secretion of matrix metalloproteases, and severe inflammation determines the fate of the plaque and the risk of sudden events. Various chronic infectious (e.g., periodontitis) and inflammatory diseases (e.g., rheumatoid arthritis, systemic lupus erythematosus, and psoriasis) are significant risk factors for cardiovascular events [11,12]. 

Periodontal disease is one of the most prevalent inflammatory diseases worldwide and its association with hypertension and atherosclerosis has already been demonstrated [13,14]. The prevalence of periodontitis is approximately 50% and it affects approximately 11.2% of the world’s population: 21% have gingivitis, 3.9% have shallow periodontal pockets, and 0.8% have deep periodontal pockets [15]. It is the sixth most prevalent disease in humans [16]. The prevalence and exacerbation of periodontal disease increase with age [15,17]. The pathophysiology of periodontitis involves an immunological and inflammatory response that induces dysregulation in the host in the presence of periodontal bacteria [18]. Periodontitis is also associated with higher serum levels of inflammatory biomarkers such as interleukin (IL)-6/17, prostaglandin, and C-reactive protein [19]. Periodontitis is characterized by a pathological shift toward dysbiosis within the oral microbiome [20]. Dysbiosis in the oral microbiome and complex changes in its composition could influence systemic inflammatory diseases such as CVDs and their outcomes [21]. 

Research has hinted towards an association between oral disease and CVD for more than a century [22,23]. Previous epidemiological studies reported a positive association between periodontitis and CVD [24,25]. A large cohort study and a systematic review recently demonstrated a positive graded association between periodontitis and an increased risk of CVD [26,27]. Several studies have shown that risk factors—including age, smoking, and diabetes—are common in periodontal disease and atherosclerotic vascular disease. There has been an increase in the number of studies reporting that timely and regular periodontal treatment can prevent atherosclerotic vascular disease [28,29]. 

However, some short-term studies reported that although periodontal treatment resulted in a reduction in endothelial dysfunction and systemic inflammation, there was no evidence that periodontal treatment prevents atherosclerotic vascular disease or improves its prognosis [17]. So far, there is no clinical evidence suggesting that preventing periodontitis with periodontal treatment improves the clinical outcomes of CVD. Therefore, the Task Force of the European Society of Cardiology and Other Societies on Cardiovascular Disease Prevention in Clinical Practice has not yet suggested periodontal treatment to be indispensable for cardiovascular health [30]. 

Similarly, the reported relationship between CVD and periodontal disease is currently controversial. Most of the studies did not adjust for various factors such as financial income and nutritional status. In addition, very few studies have identified an association between CVD and periodontal disease in a large Korean population data set. The association between the two different diseases may also differ depending on the regional and ethnic background. Therefore, the aim of this cross-sectional study was to investigate the associations between periodontitis and CVD using the Korean Genome and Epidemiology Study Health Examinee (KoGES HEXA) data by reviewing and discussing the role of the oral microbiome in periodontitis and CVD.

## 2. Materials and Methods

### 2.1. Study Population and Data Collection

The ethics committee of Hallym University (2019-02-020) approved the use of this data. The requirement for written informed consent was waived by the Institutional Review Board. This prospective cohort study relied on the data obtained from the KoGES between 2004 and 2016. A detailed description of this data has been provided in previous studies [31,32]. We used the KoGES HEXA data of urban participants aged ≥40 years from the KoGES Consortium. It consisted of base data from 2004–2013 and follow-up data from 2012–2016.

### 2.2. Participant Selection

**Figure 1:**
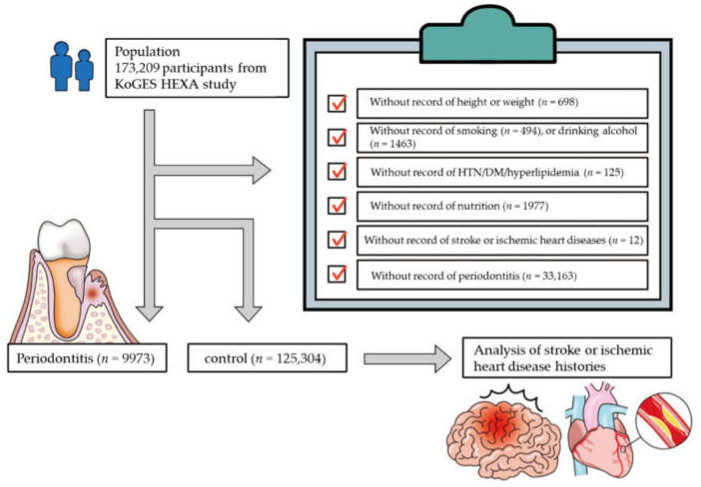
Schematic illustration of the participant selection process in this study. From a total of 173,209 participants, 9973 patients with periodontitis and 125,304 controls were selected. HTN: hypertension, DM: diabetes mellitus.

We excluded participants whose height or weight (*n* = 698); smoking history (*n* = 494); alcohol consumption (*n* = 1463); history of hypertension, diabetes mellitus, or hyperlipidemia (*n* = 125); nutrition records (*n* = 1977); history of stroke or ischemic heart disease (*n* = 12); and periodontitis (*n* = 33,163) data were missing from the records of the total 173,209 participants. Several participants were excluded as their history of periodontitis was not surveyed between 2004 and 2006. Finally, 9973 patients with periodontitis and 125,304 controls (no history of periodontitis) were selected for inclusion in the present study (Figure 1). We subsequently analyzed the history of cerebral stroke or ischemic heart disease in patients with periodontitis and the controls.

### 2.3. Survey

The participants were questioned about the history of hypertension, diabetes mellitus, hyperlipidemia, cerebral stroke (hemorrhagic or ischemic), ischemic heart disease (angina or myocardial infarction), and periodontitis by trained interviewers [33]. Body mass index (BMI) was calculated as kg/m^2^ using the health examination data. Participants were categorized as non-smokers (<100 cigarettes throughout life), former smokers (quit more than a year ago), and current smokers, according to the history of smoking; and non-drinkers, former drinkers, and current drinkers according to their alcohol consumption. Their nutritional intake [total calorie (kcal/day), protein (g/day), fat (g/day), and carbohydrate (g/day) consumption] was surveyed using a food-frequency questionnaire, which was validated by a previous study [34]. Income groups were categorized as non-respondent, low income (<~$2000 per month), middle income (~$2000–$3999 per month), and high income (~≥$4000 per month) based on the household income.

### 2.4. Statistical Analysis

The Chi-square test was used to compare the effect of sex, income group, hypertension, diabetes mellitus, dyslipidemia, smoking, and alcohol consumption on the risk of stroke and ischemic heart disease. The independent *t*-test was used to compare the effect of age, BMI, and nutritional intake. 

A logistic regression model was used to calculate the odds ratio (OR) of periodontitis for stroke/ischemic heart disease. Crude models and those adjusted for age, sex, income group, BMI, smoking, alcohol consumption, hypertension, diabetes mellitus, hyperlipidemia, and nutritional intake (total caloric, protein, fat, and carbohydrate intake) were used. Subgroup analyses for age were divided into four groups of 40–49, 50–59, 60–69, and 70–79-year-olds.

Two-tailed analyses were conducted. *p*-values less than 0.05 were considered statistically significant. All statistical analyzes were performed using SPSS v. 24.0 (IBM, Armonk, NY, USA).

## 3. Results

The general characteristics of patients with periodontitis differed from those of the controls (Table 1). 

The adjusted OR (aOR) of periodontitis for stroke was 1.35 [95% confidence interval (CI) = 1.16–1.57, *p* < 0.001]. The results of subgroup analysis for age and sex were consistent. The aOR was 1.20 (95% CI = 0.69–2.08) in the 40–49 year-old group, 1.39 (95% CI = 1.09–1.78) in the 50–59 year-old group, 1.34 (95% CI = 1.10–1.64) in the 60–69 year-old group, 1.26 (95% CI = 1.02–1.55) in men, and 1.47 (95% CI = 1.19–1.82) in women (all *p* < 0.05) (Table 2).

The crude and adjusted odd ratios (95% confidence interval) for stroke were analyzed with the factors, smoking, alcohol consumption, and the history of diabetes, hypertension, and dyslipidemia, for both the periodontitis and control groups. The analysis results for each of the factor was consistent (Table S1).

The aOR of periodontitis for ischemic heart disease was 1.34 (95%% CI = 1.22–1.48, *p* < 0.001). The results were consistent for the subgroups created based on age and sex. The aOR was 1.46 (95% CI = 1.06–2.01) in the 40–49 year-old group, 1.34 (95% CI = 1.13–1.57) in the 50–59 year-old group, 1.32 (95% CI = 1.15–1.51) in the 60–69 year-old group, 1.29 (95% CI = 1.12–1.48) in men, and 1.41 (95% CI = 1.22–1.62) in women (all *p* < 0.05) (Table 3). 

The crude and adjusted odd ratios (95% confidence interval) for ischemic heart disease were analyzed with factors, smoking, alcohol consumption, and history of diabetes, hypertension, and dyslipidemia, for both the periodontitis and control groups. The analysis results for each of the factor was consistent (Table S2).

## 4. Discussion

**Figure 2:**
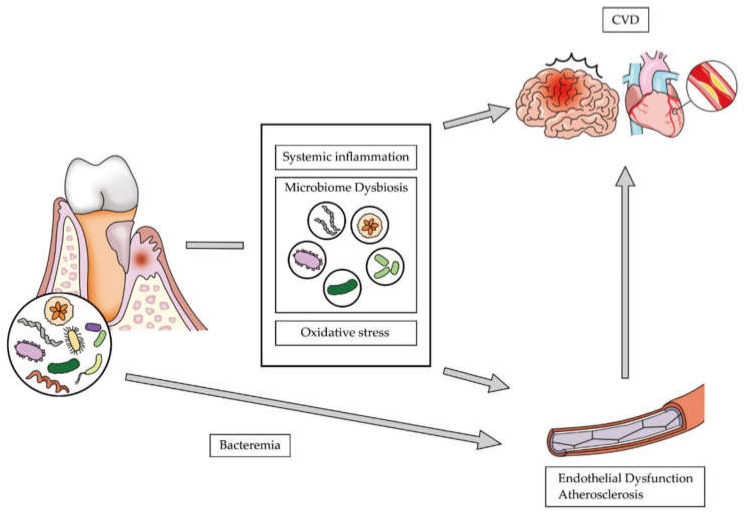
Pathophysiological theories on the association between periodontitis and cardiovascular disease.

Various pathophysiological theories have hypothesized an association between periodontitis and CVD (Figure 2). Several recent reports have focused on microbiome dysbiosis as a major factor in the etiology of CVD [35,36,37]. Oral microbiota could possibly enter into the blood circulation and cause bacteremia during routine daily activities (e.g., chewing, biting, flossing, and toothbrushing) and professional management (e.g., scaling, extraction of teeth, and periodontal probing). A systemic review, which linked the possibility of bacteremia with the periodontal condition, revealed that the risk of bacteremia increased with gingival inflammation [38]. This bacteremia is the direct putative mechanism underlying periodontitis-induced atherosclerosis [35,37]. Previous studies have shown that oral bacteria were present in the atherothrombotic tissues in patients with periodontitis and that there might be a higher positive association between bacteria associated with periodontitis and those associated with coronary plaques [39,40].

Recent studies have shown that oral microbiome dysbiosis of pathogens such as *Porphyromonas gingivalis, Tannerella forsythia, Fusobacterium nucleatum*, and *Treponema denticola*, as well as the polymicrobial infection of these microbiotas, induced the formation of the aortic toll-like receptor (TLR) along with inflammatory signaling and oxidative stress reactions in the endothelial cells [36,41,42]. There is other evidence of vascular wall inflammation and intracellular entry of periodontal bacteria such as *P. gingivalis* and *Aggregatibacter actinomycetemcomitans* [43]. Previous studies revealed that *P. gingivalis* aggravated atherothrombotic lesions, and bacterial strains expressing *P. gingivalis* hemagglutinin A, exhibited a higher ability to adhere to and invade the endothelial cells of the coronary arteries [44,45]. Other studies showed that *Eikenella corrodens* was present in the atherosclerotic area of patients with chronic periodontitis as well as in atheromatous plaques of patients with CVD and periodontitis [43,46]. Serum IL-6 levels were elevated, while those of IL-4/18 decreased in patients with periodontitis [19]. Periodontal treatment reduced the serum levels of IL-6. Peripheral neutrophils secrete excessive IL-1β, IL-8, IL-6, and tumor necrosis factor-α in patients with periodontitis. Periodontal bacteria could induce the formation of antibodies that can activate monocytes, cytokine production, and endothelial cells. These antibodies decrease after periodontal treatment in patients with periodontitis. Another study reported that higher serum immunoglobulin (Ig) G levels against *P. gingivalis* linked periodontitis and CVD [47]. 

The term ‘oral microbiota’ refers to the heterogeneous group of microbial species colonizing the entire oral cavity surfaces. Approximately 700 bacterial species have been discovered in the oral cavity and up to 35% of them have not yet been cultured. Oral microbiota may be involved in the development of primary tumors outside of the head and neck region [48,49]. Oral microbiome dysbiosis causes systemic inflammation and induces the production of proinflammatory cytokines, which can activate inflammatory pathways and recruit dedicated immune cells. This may be the indirect mechanism underlying periodontitis-induced atherosclerosis and CVD [35,36,37]. 

One study reported significantly higher levels of fibrinogen in patients with both periodontitis and CVD compared to those with only one of the two diseases [50]. Periodontal treatment significantly decreased the level of fibrinogen [51,52]. Previous studies demonstrated the association between higher platelet activation and periodontitis, which may be reversed by periodontal treatment [53]. 

Oxidative stress was also described in the pathogenesis of coronary atherosclerotic diseases [54,55]. Similarly, different studies reported that periodontitis was associated with excessive reactive oxygen species production or elevated oxidative injury to periodontal tissues [56]. The increased oxidative stress was explained by evaluating the fibroblasts in patients with periodontitis. Bacterial lipopolysaccharides induced oxidative stress and mitochondrial dysfunction by limiting mitochondrial expression/mass and membrane potential [56]. 

This study discovered a significant association between periodontitis and CVD (stroke and ischemic heart disease) in all ages and sexes with the help of the KoGES HEXA data. The result was similar to previous studies that evaluated the association between periodontitis and CVD. 

Aging could also be an important factor that affects the dynamics between periodontitis and CVD. However, this study demonstrated the association between periodontitis and CVD after excluding these influential factors. This study showed that periodontitis significantly affected CVD across all ages and sexes, even after adjusting for various factors including aging, sex, BMI, income, smoking, alcohol consumption, hypertension, diabetes, and hyperlipidemia (Table 1). This study endeavored to include other influential factors that could impact the association between periodontitis and CVD. This study adjusted for many factors and used a large population data. Nevertheless, there were a few limitations. First, the data collected by a questionnaire survey could be subjective and inaccurate, compared to the data collected by dentists or doctors. Second, it was impossible to include all the influential factors in a single study. Instead, this study adjusted as many factors as possible to minimize the surveillance bias. Finally, the pathophysiological relationship between the two diseases could not be confirmed in this study.

## 5. Conclusions

Periodontitis is associated with CVD and oral microbiome dysbiosis might play a significant role in periodontitis-induced atherosclerosis and CVD. Periodontitis may be a risk factor that affects the development of CVD and therefore could have its risk reduced by proper management and prevention. However, there are several contentious opinions and studies regarding this, therefore, further studies are required to elucidate the association between periodontal treatment and CVD.

## Figures and Tables

**Table 1:** General characteristics of the participants.

Characteristics	Total Participants		*p*-Value
	Periodontitis	Control	
Age (mean, SD, y)	54.8 (7.9)	53.0 (8.3)	<0.001 ^†^
Age group (*n*, %, y)			<0.001 *
40–49	2650 (26.6)	46,132 (36.8)	
50–59	4332 (43.4)	48,796 (38.9)	
60–69	2772 (27.8)	28,215 (22.5)	
70–79	219 (2.2)	2161 (1.7)	
Sex (n, %)			<0.001 *
Men	3851 (38.6)	43,400 (34.6)	
Women	6122 (61.4)	81,904 (65.4)	
BMI (mean, SD, kg/m^2^)	24.0 (2.9)	23.9 (2.9)	<0.001 ^†^
Income (n, %)			<0.001 *
Missing, no response	765 (7.7)	10,849 (8.7)	
Lowest	3437 (34.5)	35,580 (28.4)	
Middle	3675 (36.8)	49,421 (39.4)	
Highest	2096 (21.0)	29,454 (23.5)	
Smoking status (*n*, %)			<0.001 *
Nonsmoker	6689 (67.1)	91,108 (72.7)	
Past smoker	1795 (18.0)	18,591 (14.8)	
Current smoker	1489 (14.9)	15,605 (12.5)	
Alcohol consumption (*n*, %)			<0.001 *
Non-drinker	4787 (48.0)	64,024 (51.1)	
Past drinker	479 (4.8)	4536 (3.6)	
Current drinker	4707 (47.2)	56,744 (45.3)	
Hypertension (*n*, %)	2771 (27.8)	28,068 (22.4)	<0.001 *
Diabetes mellitus (*n*, %)	1176 (11.8)	9812 (7.8)	<0.001 *
Hyperlipidemia (*n*, %)	2204 (22.1)	17,485 (14.0)	<0.001 *
Nutritional intake (mean, SD)			
Total calories (kcal/d)	1760.2 (580.5)	1749.5 (569.4)	0.069
Protein (g/d)	58.9 (26.6)	59.8 (26.4)	0.002 ^†^
Fat (g/d)	27.5 (18.5)	28.3 (18.2)	<0.001 ^†^
Carbohydrate (g/d)	315.0 (95.2)	309.8 (92.8)	<0.001 ^†^
Stroke (*n*, %)	207 (2.1)	1523 (1.2)	<0.001 *
Ischemic heart disease (*n*, %)	494 (5.0)	3756 (3.0)	<0.001 *

* Chi-square test. Significance set at *p* < 0.05; ^†^ Independent *t*-test. Significance set at *p* < 0.05. BMI: body mass index.

**Table 2:** Crude and adjusted odd ratios (95% confidence interval) for stroke in the periodontitis and control groups.

Characteristics	Odd Ratios for Stroke			
	Crude	*p*-Value	Adjusted ^†^	*p*-Value
Total participants (*n* = 135,277)				
Periodontitis	1.72 (1.49–2.00)	<0.001 *	1.35 (1.16–1.57)	<0.001 *
Control	1.00		1.00	
Age 40–49 years old (*n* = 48,782)				
Periodontitis	1.47 (0.85–2.54)	0.167	1.20 (0.69–2.08)	0.528
Control	1.00		1.00	
Age 50–59 years old (*n* = 53,128)				
Periodontitis	1.57 (1.23–2.01)	<0.001 *	1.39 (1.09–1.78)	0.009 *
Control	1.00		1.00	
Age ≥ 60 years old (*n* = 33,367)				
Periodontitis	1.49 (1.22–1.81)	<0.001 *	1.34 (1.10–1.64)	0.004 *
Control	1.00		1.00	
Men (*n* = 47,251)				
Periodontitis	1.53 (1.25–1.88)	<0.001 *	1.26 (1.02–1.55)	0.031 *
Control	1.00		1.00	
Women (*n* = 88,026)				
Periodontitis	1.85 (1.50–2.29)	<0.001 *	1.47 (1.19–1.82)	<0.001 *
Control	1.00		1.00	

* Logistic regression model; Significance at *p* < 0.05.; ^†^ Models adjusted for age, sex, income group, body mass index, smoking, alcohol consumption, hypertension, diabetes mellitus, hyperlipidemia, and nutritional intake (total caloric, protein, fat, and carbohydrate consumption).

**Table 3:** Crude and adjusted odd ratios (95% confidence interval) for ischemic heart disease in the periodontitis and control groups.

Characteristics	Odd Ratios for Ischemic Heart Disease			
	Crude	*p*-Value	Adjusted ^†^	*p*-Value
Total participants (*n* = 135,277)				
Periodontitis	1.69 (1.53–1.86)	<0.001 *	1.34 (1.22–1.48)	<0.001 *
Control	1.00		1.00	
Age 40–49 years old (*n* = 48,782)				
Periodontitis	1.87 (1.37–2.56)	<0.001 *	1.46 (1.06–2.01)	0.020 *
Control	1.00		1.00	
Age 50–59 years old (*n* = 53,128)				
Periodontitis	1.51 (1.29–1.78)	<0.001 *	1.34 (1.13–1.57)	0.001 *
Control	1.00		1.00	
Age ≥ 60 years old (*n* = 33,367)				
Periodontitis	1.42 (1.25–1.62)	<0.001 *	1.32 (1.15–1.51)	<0.001 *
Control	1.00		1.00	
Men (*n* = 47,251)				
Periodontitis	1.53 (1.34–1.75)	<0.001 *	1.29 (1.12–1.48)	<0.001 *
Control	1.00		1.00	
Women (*n* = 88,026)				
Periodontitis	1.77 (1.55–2.03)	<0.001 *	1.41 (1.22–1.62)	<0.001 *
Control	1.00		1.00	

* Logistic regression model; Significance at *p* < 0.05; ^†^ Models adjusted for age, sex, income group, body mass index, smoking, alcohol consumption, hypertension, diabetes mellitus, hyperlipidemia, and nutritional intake (total caloric, protein, fat, and carbohydrate consumption).

## Supplementary Materials

The following are available online at https://www.mdpi.com/1660-4601/17/14/5237/s1, Table S1: Subgroup analyses of crude and adjusted odd ratios (95% confidence interval) for stroke in periodontitis and control groups according to smoking, alcohol consumption, and history of diabetes, hypertension, and dyslipidemia, Table S2: Subgroup analyses of crude and adjusted odd ratios (95% confidence interval) for ischemic heart disease in periodontitis and control groups according to smoking, alcohol consumption, and history of diabetes, hypertension, and dyslipidemia.
