# Supplementary material for: Cross-Sectional Analysis of the Association between Periodontitis and Cardiovascular Disease Using the Korean Genome and Epidemiology Study Data
Source: Int J Environ Res Public Health. 2020 Jul 20;17(14):5237. doi: 10.3390/ijerph17145237 (PMC7400444; doi:10.3390/ijerph17145237)
Supplement: Supplementary file 1 [file ijerph-17-05237-s001.pdf]

Supplementary Materials

Table S1. Subgroup analyses of crude and adjusted odd ratios (95% confidence interval) for stroke in periodontitis and control groups according to smoking, alcohol consumption, and history of diabetes, hypertension, and dyslipidemia.

**Characteristics**	**Odd Ratios for Stroke**			
	**Crude**	***p*-Value**	**Adjusted †**	***p*-Value**
Smoking				
Nonsmoker (n = 97,797)				
Periodontitis	1.73 (1.42–2.11)	<0.001 *	1.40 (1.15–1.71)	0.001 *
Control	1.00		1.00	
Past smoker (n = 20,386)				
Periodontitis	1.55 (1.19–2.03)	0.001 *	1.33 (1.01–1.74)	0.042 *
Control	1.00		1.00	
Current smoker (n = 17,094)				
Periodontitis	1.64 (1.10–2.45)	0.016 *	1.23 (0.82–1.86)	0.320
Control	1.00		1.00	
Alcohol consumption				
Nondrinker (n = 68,811)				
Periodontitis	1.54 (1.23–1.93)	<0.001 *	1.25 (1.00–1.57)	0.056
Control	1.00		1.00	
Past drinker (n = 5015)				
Periodontitis	1.77 (1.21–2.58)	0.003 *	1.55 (1.04–2.29)	0.030 *
Control	1.00		1.00	
Current drinker (n = 61,451)				
Periodontitis	1.82 (1.45–2.28)	<0.001 *	1.43 (1.14–1.80)	0.002 *
Control	1.00		1.00	
History of diabetes				
Non-diabetes (n = 124,289)				
Periodontitis	1.66 (1.40–1.97)	<0.001 *	1.35 (1.14–1.61)	0.001 *
Control	1.00		1.00	
Diabetes (n = 10,988)				
Periodontitis	1.48 (1.10–1.98)	0.009 *	1.39 (1.03–1.88)	0.029 *
Control	1.00		1.00	
History of hypertension				
Non-hypertension (n = 104,438)				
Periodontitis	1.64 (1.29–2.07)	<0.001 *	1.34 (1.05–1.70)	0.018 *
Control	1.00		1.00	
Hypertension (n = 30,839)				
Periodontitis	1.52 (1.25–1.83)	<0.001 *	1.38 (1.14–1.67)	0.001 *
Control	1.00		1.00	

History of dyslipidemia				
Non-dyslipidemia (*n* = 115,588)				
Periodontitis	1.69 (1.42–2.02)	<0.001 *	1.40 (1.17–1.68)	<0.001 *
Control	1.00		1.00	
Dyslipidemia (*n* = 19,689)				
Periodontitis	1.45 (1.12–1.89)	0.005 *	1.28 (0.98–1.67)	0.071
Control	1.00		1.00	

* Logistic regression model, Significance at $p < 0.05$. † Adjusted for age, sex, income group, BMI, smoking, alcohol consumption, hypertension, diabetes mellitus, hyperlipidemia histories, and nutritional intake (total calories, protein, fat, and carbohydrate intake).

Table 2. Subgroup analyses of crude and adjusted odd ratios (95% confidence interval) for ischemic heart disease in periodontitis and control groups according to smoking, alcohol consumption, and history of diabetes, hypertension, and dyslipidemia.

Characteristics	Odd Ratios for Ischemic Heart Disease			
	Crude	*p*-Value	Adjusted †	*p*-Value
Smoking				
Nonsmoker (*n* = 97,797)				
Periodontitis	1.69 (1.48 - 1.91)	<0.001*	1.37 (1.20 - 1.56)	<0.001 *
Control	1.00		1.00	
Past smoker (*n* = 20,386)				
Periodontitis	1.52 (1.27 - 1.83)	<0.001*	1.30 (1.08 - 1.57)	0.006 *
Control	1.00		1.00	
Current smoker (*n* = 17,094)				
Periodontitis	1.66 (1.29 - 2.13)	<0.001 *	1.35 (1.04 - 1.74)	0.024 *
Control	1.00		1.00	
Alcohol consumption				
Nondrinker (*n* = 68,811)				
Periodontitis	1.71 (1.49 - 1.96)	<0.001 *	1.40 (1.22 - 1.61)	<0.001 *
Control	1.00		1.00	
Past drinker (*n* = 5015)				
Periodontitis	1.38 (0.99 - 1.93)	0.058	1.20 (0.85 - 1.70)	0.292
Control	1.00		1.00	
Current drinker (*n* = 61,451)				
Periodontitis	1.69 (1.46 - 1.96)	<0.001 *	1.33 (1.14 - 1.55)	<0.001 *
Control	1.00		1.00	
History of diabetes				
Non-diabetes (n = 124,289)				
Periodontitis	1.65 (1.47 - 1.84)	<0.001 *	1.36 (1.21 - 1.52)	<0.001 *
Control	1.00		1.00	
Diabetes (*n* = 10,988)				
Periodontitis	1.39 (1.14 - 1.69)	0.001 *	1.31 (1.08 - 1.61)	0.008 *
Control	1.00		1.00	
History of hypertension				
Non-hypertension (*n* = 104,438)				
Periodontitis	1.62 (1.41 - 1.86)	<0.001 *	1.33 (1.16 - 1.53)	<0.001 *
Control	1.00		1.00	
Hypertension (*n* = 30,839)				
Periodontitis	1.53 (1.34 - 1.75)	<0.001 *	1.36 (1.19 - 1.57)	<0.001 *
Control	1.00		1.00	
History of dyslipidemia				
Non-dyslipidemia (*n* = 115,588)				
Periodontitis	1.57 (1.39 - 1.77)	<0.001 *	1.32 (1.17 - 1.49)	<0.001 *
Control	1.00		1.00	
Dyslipidemia (n = 19,689)				
Periodontitis	1.56 (1.32 - 1.84)	<0.001 *	1.41 (1.19 - 1.68)	<0.001 *
Control	1.00		1.00	

* Logistic regression model, Significance at $p < 0.05$. † Adjusted for age, sex, income group, BMI, smoking, alcohol consumption, hypertension, diabetes mellitus, hyperlipidemia histories, and nutritional intake (total calories, protein, fat, and carbohydrate intake).
